# Gender diversity in autistic clients: an ethical perspective

**DOI:** 10.3389/fpsyt.2023.1244107

**Published:** 2023-09-20

**Authors:** Katie Jo Glaves, Leah Kolman

**Affiliations:** ^1^Protea Wellness, Seattle, WA, United States; ^2^Department of Couple and Family Therapy, Antioch University, Seattle, WA, United States

**Keywords:** autism, gender dsyphoria, gender-affirming care, ethics, autonomy

## Abstract

Autonomy and dignity are key ethical principles in psychiatric and psychological codes of ethics. Yet, when working with autistic individuals who are transgender/gender non-conforming (TGNC), non-autistic and cisgender clinicians can unintentionally take away client dignity and autonomy by disbelieving or stigmatizing clients’ gender identities. Lack of awareness or discomfort around autism and gender dysphoria can lead clinicians to assumptions and interventions that damage both client rapport and client mental health; discouraging clients from being honest with clinicians about their mental health, and potentially leading to harm. Clinicians must take an intersectional view of their autistic clients’ gender identities to reduce stigma and recognize the needs of the whole person. Facilitating access to gender-affirming care is an important part of caring for TGNC clients, including those who are autistic. The authors will discuss the ethical imperative to help autistic clients access gender-affirming care, while discussing common concerns clinicians have when helping autistic clients access this care, as well as the need to believe and support autistic clients when they share their gender identities with clinicians.

## 1. Introduction

Multiple studies have shown that autistic individuals are more likely than the general populace to experience gender dysphoria (1–3). In this paper, we will use the term ‘transgender and gender non-conforming’ (TGNC) to encompass individuals whose gender identities differ from those assigned at birth. This term includes transgender, non-binary, two-spirited, and other non-cisgender identities. The co-occurrence of TNGC identities and autism spectrum disorder presents a challenge to clinicians as guidance related to working with TNGC, autistic individuals is still being created and debated; clinical consensus is forming, but is not well established (4).

## 2. Current climate and impact on providers

Many discussions about TGNC individuals and autism do not address a clinician’s ethical duties to clients; this is complicated by the increase in threats to clinicians’ abilities to treat TGNC children and adults in many states; as laws pass that ban gender-affirming care, many clinicians may be increasingly reluctant to provide gender-affirming care for autistic individuals, as doing so is potentially more complex than providing care to non-autistic individuals and might subject clinicians to legal action or ethical complaints.

An emergency order issued by Missouri’s Attorney General (5) specifically bans providing gender-affirming care to patients until they have received “comprehensive screening” to determine if the client is autistic; this implies that if the patient is autistic, that treatment might be restricted or different than treatment for a non-autistic individual, raising ethical questions for providers, as well as concerns for potential loss of license when treating TGNC, autistic clients.

Arkansas’ law attempting to restrict gender-affirming care for minors, now struck down, attempted to restrict access to gender-affirming care for children with any concurrent mental health conditions, explicitly including autism (6). Georgia’s Senate Bill 140 (7), includes in its rationale for banning gender-affirming care that “gender dysphoria is often comorbid with other mental health and developmental conditions, including autism spectrum disorder,” which heavily implies the co-occurrence must be due to either over-diagnosis or some form of pathology.

In this difficult political climate, with spotty or inconclusive guidance, what is appropriate for a clinician to do when an autistic individual presents with gender dysphoria? We would suggest looking to ethical principles to guide us. The ethical principle of autonomy holds “that all persons have intrinsic and unconditional worth, and therefore, should have the power to make rational decisions and moral choices, and each should be allowed to exercise his or her capacity for self-determination (8). This remains true, even for clients who have developmental or intellectual disabilities; indeed, the onus is on us, as professionals, to make sure we are fostering autonomy in persons with developmental or intellectual disabilities (9).

Further, without paying attention to the inherent dignity of each client, we encourage shame, which harms their mental health. If we as clinicians see autism as a culture, as many writers suggest we should (10–12), we must understand gender dysphoria as something that intersects with autism spectrum disorder, as the APA recommends clinicians understand how gender identity and culture intersect (13). Paying attention to the culture of a client restores dignity, as the client feels understood by their clinician (14).

## 3. Assumptions and concerns

When discussing gender dysphoria in autistic individuals, many writers express concerns that gender may be a passing special interest or obsession for their autistic clients (15, 16) or assume the clients are experiencing identity confusion due to social struggles (17–19). These concerns and assumptions seem to be rooted in stigma and the belief that either autistic clients cannot be genuinely TGNC or that the gender concerns must be part of another mental health disorder.

Clinicians often begin by treating gender dysphoria in autistic people as though it will pass, given time. However, in several case studies, the client’s gender dysphoria did not pass (20–23). Autistic individuals who are TGNC report being told by parents, professionals and others that their desire to be another gender is a passing obsession (24), even when the individuals in question feel certain about who they are. In one case study (15), gender dysphoria lasted multiple years; the writer takes it as a victory that, in time, after multiple years, the individuals did stop asking to transition, although after reading the case report, one wonders if the clients just gave up in exhaustion, due to constant invalidation. In sum, in most case studies, gender dysphoria was not transitory.

A few case studies attempted to ‘correct’ children who endorsed being a different gender than the one assigned at birth through behavioral treatment or medication; Janssen et al. (20) report in a case study in which “dress in a masculine manner” was included in a treatment plan for a client who was assigned male-at-birth; despite the client’s clear preference for feminine clothing and desire to be seen as female. In this case, attempts at behavioral correction caused the client distress while shifting to gender-affirming care helped the client feel comfortable in her body. Another case study (21) describes similar, unsuccessful, attempts to change gendered behavior using behavioral principles. Attempts to use behavioral principles to shift gender-related behavior is a form of conversion therapy that is proven to be harmful (25).

Notably, Applied Behavioral Analysis (ABA), a form of autism treatment associated with trauma symptoms in autistic people (26, 27), was created by Ole Ivar Lovaas, who was also a proponent of conversion therapy for LGBTQ people (28). Lovaas was involved in the “Feminine Boy Project” where one of Lovaas’ graduate students attempted to “correct” children’s “disturbed” gender expression; one participant committed suicide in adulthood and his family blamed his suicide on participation in the experiment (28, 29). Many autistic self-advocates and queer disability theorists highlight the way both ABA and LGBTQ conversion therapy disregard the “possibility of following the needs, wants, or inner experiences and desires of children labeled autistic or gender “disturbed.” (30) Attempts to behaviorally “correct” gender should be viewed with suspicion for many reasons, including this disturbing history.

Landen & Rasmussen (31) and Perera (32) describe two separate cases of gender identity concerns in autistic children; both initially saw the gender identity concerns as part of OCD, but when treated with medication, other OCD symptoms receded, but the gender identity concerns remained. This suggests that defaulting to see gender identity as relating to OCD may not be an effective path to treatment.

The idea that social struggles could underlie gender identity struggles is intriguing. An autistic person might struggle socially and conclude that they are not the gender they were assigned at birth. In one case (19), social communication rehabilitation did temporarily stop requests for gender-affirming care, but the client still ultimately returned to requesting gender-affirming care. A clinician should, with careful questioning, be able to discern the difference between magical thinking that transition will resolve a client’s social struggles and a realistic appraisal of the risk and benefits of gender transition.

## 4. Ethical context to objections

While many concerns about the authenticity of gender dysphoria in autistic clients come out of a place of care, they inadvertently ignore ethical concerns related to client autonomy and dignity. Indeed, it is striking how rarely ethical concerns are raised. One case study (33) states unambiguously that the clients “retain the right to self-determination.” Some other articles do state autism should not be a rule-out for gender-affirming care, but without clear emphasis on ethics.

One anticipated objection to providing gender-affirming care is that clinicians also have the ethical duty to not cause harm. Given that some gender-affirming medical treatments do have risks as well as benefits, is leaving a client’s gender dysphoria untreated the right choice? We would argue no, as untreated gender dysphoria raises the risk of suicide for clients and causes other, negative, mental health impacts, whereas gender-affirming care reduces the risk of suicide and negative mental health outcomes in the long and short term (34–37).

In addition, even if a clinician sees providing gender-affirming care as ‘risk,’ what is our right to decide what risks a client takes on? Some writers argue persuasively for the dignity of risk (38, 39): to allow clients to take on self-chosen, well-understood risk respects both their autonomy and their dignity and reduces stigma and shame. We remove dignity when we ‘protect’ clients from risks and we violate our ethical principles. Autistic clients themselves understand this; in a qualitative study (40), autistic, gender-diverse participants discussed the distress and indignity of having to rely on external supports which invalidate and question the client’s right to make their own decisions due to the client’s autism. Autistic clients should retain the same rights to autonomy and the same human dignity as non-autistic clients; to act otherwise is to perpetuate stigma.

## 5. Adopting a new lens

All clinicians could benefit from utilizing a perspective toward autistic and TGNC people similar to the perspective shared with many new therapists: stay curious and collaborate with the client. The context of a client’s life and how they have come to their perspective can be just as important as the perspective itself.

If a clinician has questions about the validity of an autistic client’s gender identity, then in the interest of maintaining client autonomy, clinicians must first ask themselves why they question that validity. Does the client’s feelings about gender distress the client or distress the clinician? Are we helping the client investigate, or are we pushing them in a certain direction? Self-doubt can be a common topic for TGNC people for a variety of reasons, and it’s vital to a client’s autonomy and dignity that clinicians provide support or guidance without pushing them in any one direction.

Self-doubt of gender identity often occurs for a variety of reasons, and can often be a symptom of anxiety or depression related to untreated dysphoria, public stigmatization, or a lack of community support. Research related to internalized oppression corroborates that stigmatization and oppression often lead people to doubt themselves; one study (41) shows that autistic persons may be especially susceptible to internalized ableism and especially sensitive to stigma. Experiencing ‘double minority’ status as both autistic and gender-diverse almost certainly takes a toll on self-confidence. Strang et al.’s (24) study reports that many autistic, TGNC youth “are at risk for being misunderstood in terms of their gender and gender needs;” clinicians should take special care with this population to seek to understand first. Clinicians who discourage gender identity exploration may inadvertently contribute to these internalized feelings. We believe clinicians best maintain client autonomy by helping guide the client through their feelings, and supporting them to find the answer that’s right for their specific circumstances.

It is also important for clinicians to maintain flexibility as they work with TGNC and autistic clients. Standard practices to alleviate anxiety may be less effective when a client’s community or support system holds hostile views towards non-cisgender people, or when the client has had bad experiences with previous clinicians. Clients may hold back information in initial sessions, including their questioning of gender identity, until they can see that a clinician is safe to disclose to; clinicians can stigmatize clients by seeing this as insecurity in their choice, rather than as the client assessing the clinician before deciding if the clinician is safe. Clinicians can also signal their willingness to talk about gender by asking about pronouns and gender identity in intake documents and by not assuming a client is cisgender.

Clinicians also need to see autism as a culture; TNGC identities occurs within the cultural framework of autism. Autistic voices are just now ‘joining’ the academic discussion, to the joy of some and the frustration of others (42). With the increased focus on autism as an identity, not just a disorder, and the high concurrence of autism and TNGC identities, clinicians need to understand an autistic client’s gender identity as part of their culture and make an effort to understand the norms of autistic culture, which often includes more space for non-cisgender identities. That does not, of course, remove our obligations for appropriate assessment and diagnosis, but we cannot discount autistic culture without discounting the dignity of our clients. We must cultivate cultural humility when approaching autistic culture, just as we would with any other culture.

We believe most clinicians would agree that our clients all come to see us with personal skills they have developed to navigate their lives. We must recognize that autistic TGNC clients also come with unique skills, and we encourage clinicians to actively affirm these skills throughout their work with their clients. Affirmation of self-advocacy skills, boundary setting, and, simply, believing the client, can be particularly bolstering for autistic TGNC clients learning to trust themselves and how they view the world. These clients will often experience less anxiety when they learn how to listen to and trust their instincts. We also view a client investigating their own symptoms to improve their own lives as an essential life skill that should be encouraged; this is the epistemic humility that Chapman and Botha (43) name as a key to doing neurodivergence-informed psychotherapy. This humility is a willingness to see the dignity and autonomy of autistic individuals and believe them when they share about their lives and perspectives, about gender and about autism. We affirm their dignity when we believe.

## 6. Considerations relating to TNGC, autistic children

Working with autistic TGNC children and teens requires navigating between a client’s need for autonomy and potential parental anxiety about allowing a child or teen that autonomy. Younger clients may feel more pressure to stop or deny their gender questioning, or sound unsure of themselves discussing the topic, if they live in a home they perceive to be unsupportive; conversely, young children who socially transition and have supportive parents show no elevations in depression (35). One study of homeless or at-risk LGBT youth (44) found that LGBT youth made up 40% of their servicers’ clientele and that 68% of those LGBT youth reported experiencing family rejection. Youth in a study (24) reported that “gender diversity obfuscated their ASD in the eyes of others due to common misunderstandings of what constitutes autism,” which can lead to problematic reactions from others and distress in the client. A client assigned male at birth who transitions now faces, in addition to the struggles of being TGNC, the struggles of autistic women and girls, who often report high pressure to conform to gendered norms and greater judgment from female peers if they fail to meet those norms due to their autism (45).

We believe the best approach to building trust with these clients may be to show flexibility in places where, as a profession, we are often too rigid and fail to take into account the impact of autism on a client’s narrative. If a client struggles to answer hypothetical questions or to imagine future scenarios, clinicians might find other ways to ask their questions; for example, the technique of asking a client to draw themselves was an excellent adaptation for an intellectually-disabled, autistic minor client in one study, yielding useful information (46).

Clinicians working with these populations could also benefit by finding and maintaining information sources based in those communities. A study argued that youth and parents could both benefit from hearing stories of and feeling connections to other TNGC people (47) and we concur with that assessment. We believe clinicians who take in information from TGNC communities will better serve their clients overall.

Parental involvement can often be crucial to a minor’s mental health. When it comes to autism and gender, many parents are unsure whose advice to trust. Some parents may present with curiosity and desire to learn, while others may present as doubting of, or hostile to, their child’s gender identity based on public information they have been given and the (lack of) support they have been offered. Kuvalanka (48) discusses ways in which parents feel supported and not supported by the larger systems in their children’s lives; the importance of clinicians who can help families navigate systems cannot be overstated.

If parents express hostility and doubt about a child’s gender identity, clinicians must offer education, referrals and support, while also believing the child in question. One of the writers of this paper (KJG) has navigated this situation multiple times; the vast majority of parents love their child and when given appropriate and kind support and information, do come to support their TGNC child in transitioning. If this does not happen, believing the child at least provides comfort and support to that child from a trusted adult.

Ehrensaft (49) proposed clinicians could work with parents to help their children navigate “gender mazes” by helping parents keep focus on their children’s needs and desires. We believe mental health professionals are in a uniquely important position to provide trustworthy, accurate information that will help parents navigate these “mazes” and support their children.

We also believe that to work with these families in an evidence-based, ethical, effective manner, clinicians should understand the substantial research available that supports gender-affirming care for youth and adults; while much of it is not yet specific to the autistic and TGNC populations, that research is emerging. Clinicians should also name for parents that we do not need complete studies of autism and gender identity to affirm their child’s gender identity; autistic children and teens are likely to respond to gender-affirming care the way non-autistic children and teens respond, and the evidence shows gender-affirming care benefits TGNC clients. A clinician who has accurate information will be better equipped to provide resources and support to parents to help them support their child and affirm that child’s autonomy and dignity.

## 7. Conclusion

Autonomy and dignity are the antithesis of stigma and shame; yet for autistic and gender-diverse populations, shame and stigma can come from both their neurotype and gender identity, harming to the client. As clinicians, we must honor the dignity and autonomy of each client, believing each client, supporting each client and helping each client make decisions that are right for that client. We do this best when we acknowledge the social context in which we are working, a social context rife with transphobia and bias that can thwart our clients’ autonomy and trample their dignity. As a profession, we can support, affirm and care for gender-diverse autistic clients by believing clients; in that way, we honor clients as autonomous, dignified human beings.

## Data availability statement

The original contributions presented in the study are included in the article/supplementary material, further inquiries can be directed to the corresponding author.

## Author contributions

All authors listed have made a substantial, direct, and intellectual contribution to the work and approved it for publication.

## Conflict of interest

The authors declare that the research was conducted in the absence of any commercial or financial relationships that could be construed as a potential conflict of interest.

## Publisher’s note

All claims expressed in this article are solely those of the authors and do not necessarily represent those of their affiliated organizations, or those of the publisher, the editors and the reviewers. Any product that may be evaluated in this article, or claim that may be made by its manufacturer, is not guaranteed or endorsed by the publisher.
